# Extirpation of the Larynx

**Published:** 1882-09

**Authors:** 


### EXTIRPATION OF THE LARYNX.

Czerny proved the possibility of extirpation of the larynx, on animals, and then Billroth executed the operation on living persons. Since that time extirpation of the larynx has been practiced about forty times, with eighteen successful results. The most severe accident in this operation, the entrance of blood into the bronchial tubes, has been avoided by, 1, lowering the head of the patient; 2, performing tracheotomy a certain time before laryngotomy. The lowering of the head is objectionable, because the parts to be operated upon are not in full view; but the obturation of the the trachea after tracheotomy is the best means to avoid an escape of blood into the bronchial tubes. Trendelenburg's canula with air-sac is doubtless the best apparatus invented for this purpose. The incision is made in the median line, from a little above the hyoid bone down to the first cartilage of the trachea. The skin and cellular tissue are divided, and the sterno-hyoids are entered without difficulty on the thyroid cartilage; then the thyroid bursa is opened. The flow of blood is insignificant, and only the crico-thyroid artery has to be ligated. Then the lateral parts of the larynx are dissected with a blunt instrument, Bottini using the thermo-cautery. The thyro-hyoid and sterno-hyoid muscles are divided on their point of insertion on the cartilage. The deeper parts are separated, first the lateral lobe of the thyroid body (corpus), and the superior thyroid artery ligated. The constrictors of the pharynx are dissected from their points of insertion, and the larynx thus liberated from the lateral and posterior parts. The larynx is then seized with a tenaculum inserted into the thyroid cartilage, the organ thrown forward and the hyo-thyroid ligament dissected. The upper laryngeal nerve and artery are divided, the latter ligated. The front part of the larynx is then isolated by cutting the inferior arteries and nerves, and at last the larynx is separated from the trachea by dissection. Some operators prefer to separate the larynx from the trachea immediately after the larynx has been isolated from the lateral parts, and to dissect from below upward, dissecting the thyro-hyoid ligament last. In cases where the cancerous masses have entered upon the pharynx, œsophagus, trachea and epiglottis, the surgeon requires great discretion. To prevent the rapid retraction of the parts surrounding the trachea, Maas and Wegner divided the cricoid muscle with Liston's pincette, leaving a space for the insertion of the artificial larynx. The operation being finished, a few sutures are applied, but primary reunion is not sought for. A large soft canula, the anterior part of which is surrounded by antiseptic gauze, is introduced from the wound into the stomach, the superior part of the trachea is closed by Trendelenburg's apparatus, which is provided with an air-sac. The wound is treated antiseptically, injections being made into and through the mouth, especially in cases where the epiglottis or tongue had been elevated in the course of the operation. Nourishment (wine, beef-tea, milk) is given through the anterior aperture of the canula. Deglutition by the mouth is only possible after the wound is covered with healthy granulations, which may be perfected after a short time. The tongue acts then, even in cases where the epiglottis had to be elevated, as an obstructor, and it prevents the food falling into the trachea. Some operations have been followed by surprising success, and the wound healed without fever or any other accident. The principal causes of death have been shock and pneumonia. The main points in the operation are, 1, preliminary tracheotomy; 2, placing of the tampon; 3, insertion of the œsophageal canula; 4, antiseptic treatment. After the wound has been healed, it is necessary to introduce an artificial larynx, Gussenbauer's newly invented instrument being the most perfect. The latter is composed of a tracheal canula, a laryngeal canula containing the vocal apparatus, and a valve forcing the air through the laryngeal canula in expiration. The amplitude of vibrations of the metallic lamella is increased by the passage of the sound into the nasal fossæ.—*Archives Général de Médecine.*

